# Supplementary figures and images for: The conserved AAA ATPase PCH-2 distributes its regulation of meiotic prophase events through multiple meiotic HORMADs in C. elegans
Source: PLoS Genet. 2023 Apr 14;19(4):e1010708. doi: 10.1371/journal.pgen.1010708 (PMC10132761; doi:10.1371/journal.pgen.1010708)

A

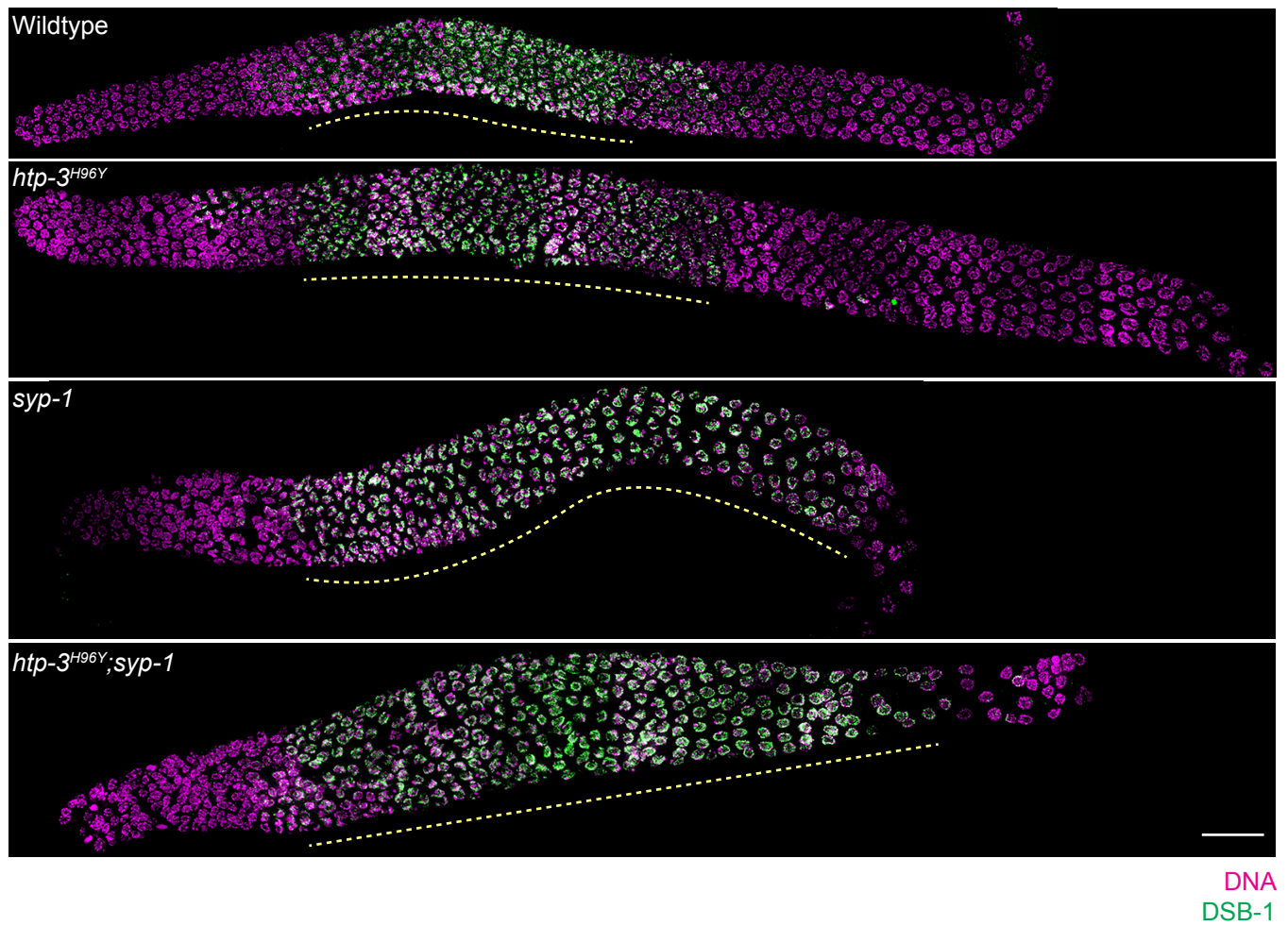

B

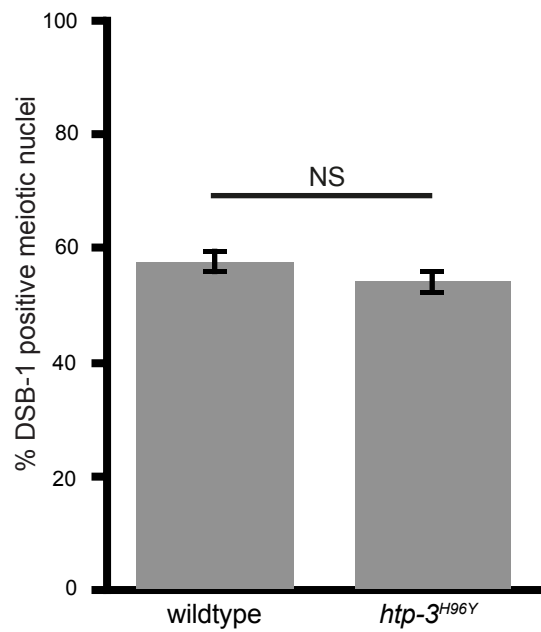

C

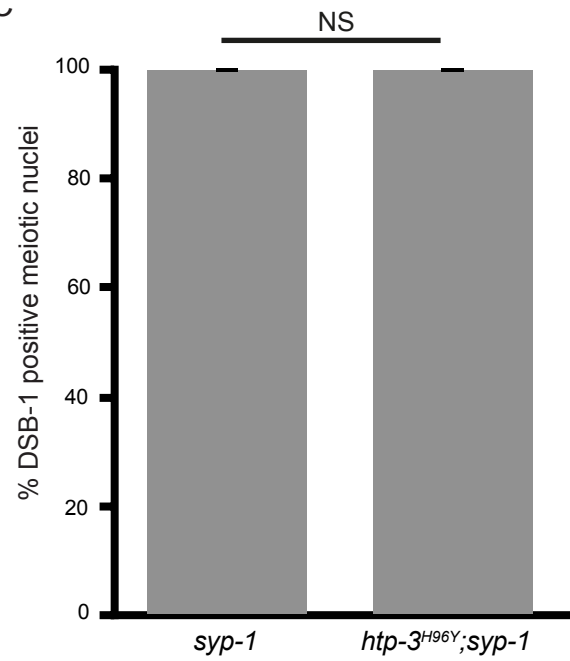

Supplement: S1 Fig — A. Full length representative germlines stained with DAPI (magenta) and DSB-1 (green). Yellow dashed line represents region of DSB-1 positive nuclei. Scalebar indicates 20 microns. B. Quantification of DSB-1 positive meiotic nuclei for wildtype (n = 1961) and htp-3 H96Y (n = 1938) mutant strains. NS indicates non-significant. C. Quantification of DSB-1 positive meiotic nuclei for syp-1 (n = 1951) and htp-3 H96Y;syp-1 (n = 1847) mutants. NS indicates non-significant. Statistical significance was assessed using two-tailed Fisher’s exact tests. Error bars represent 95% confidence intervals. (PDF) [file pgen.1010708.s001.pdf]

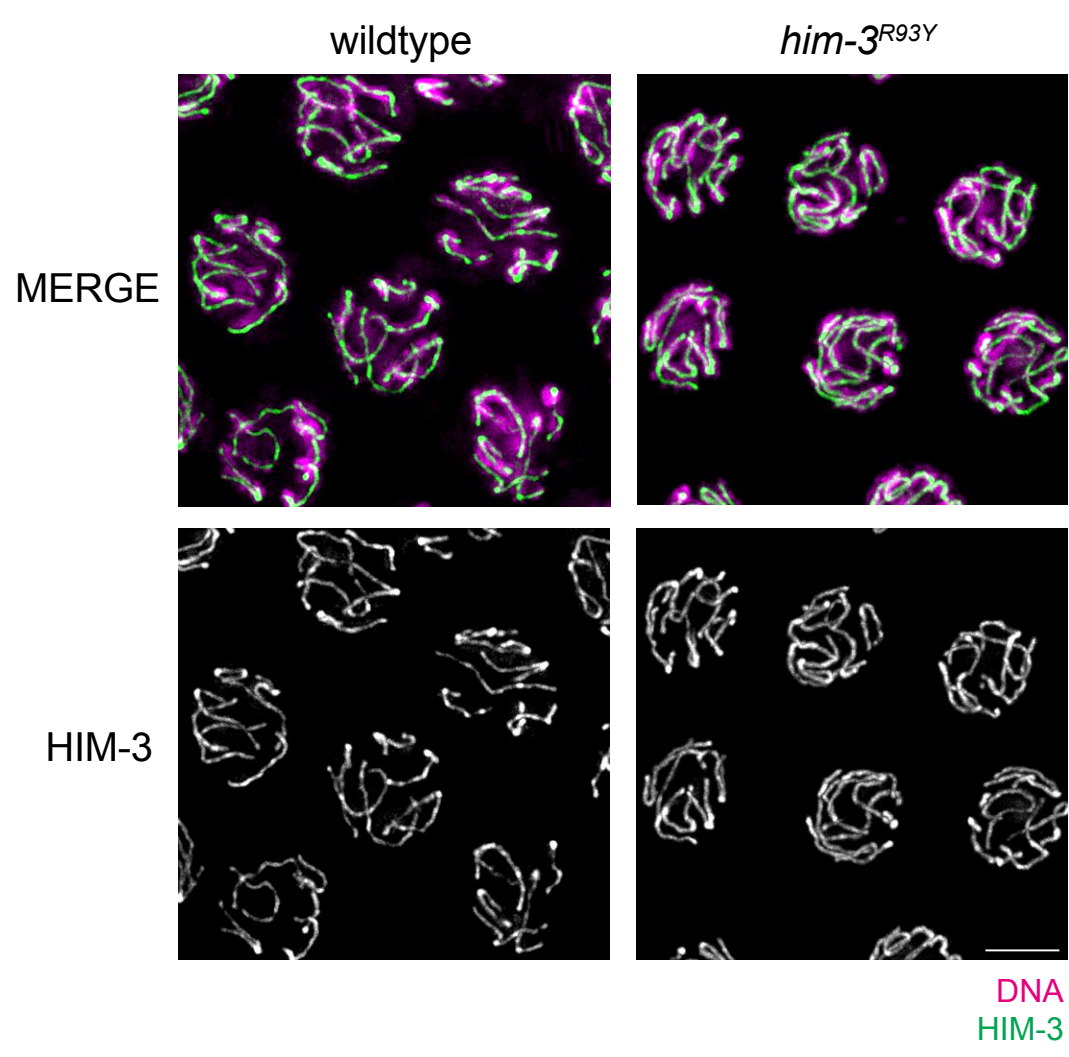

Supplement: S2 Fig — Top: Mid-pachytene nuclei from wildtype and him-3R93Y germlines stained with DAPI (magenta) and antibody against HIM-3 (green). Bottom: Mid-pachytene nuclei from wildtype and him-3R93Y germlines stained with HIM-3 (white). Scalebar indicates 5 microns. (PDF) [file pgen.1010708.s002.pdf]

A

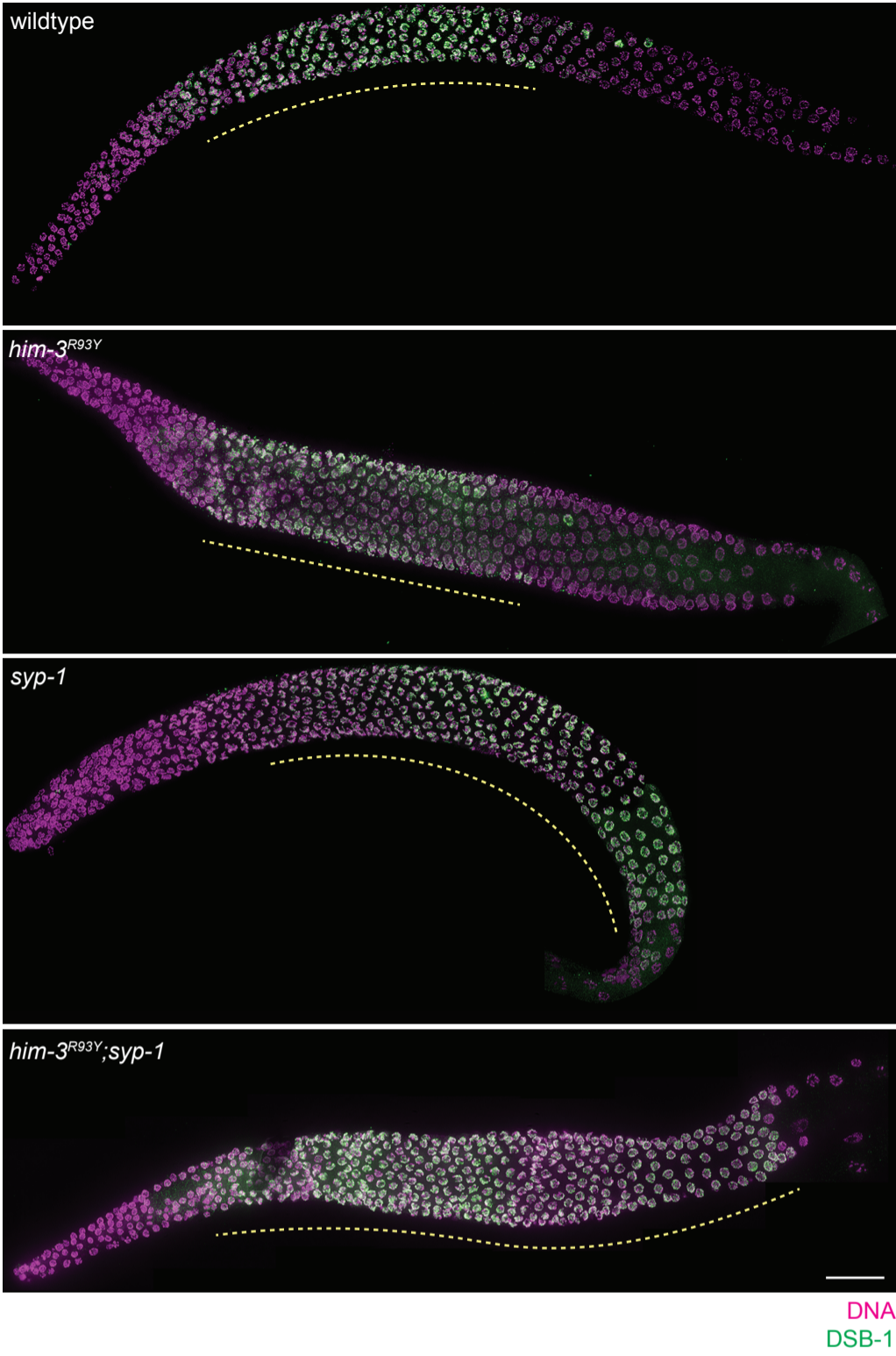

B

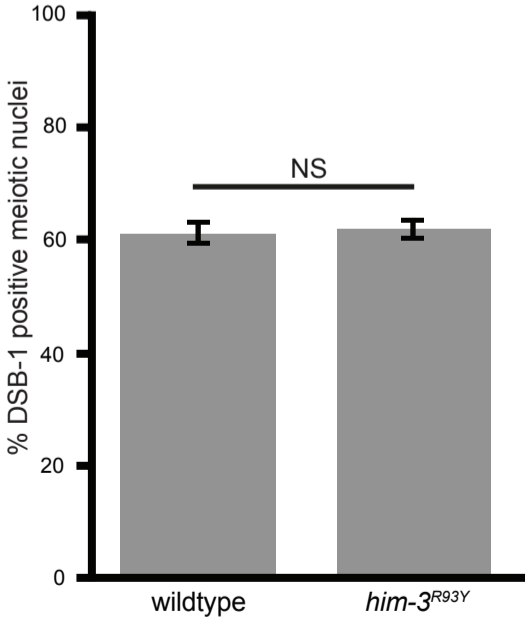

C

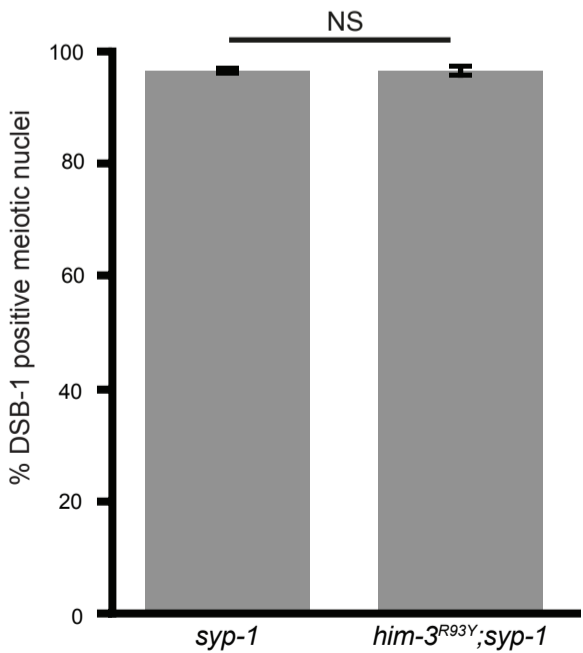

Supplement: S3 Fig — A. Full length representative germlines stained with DAPI (magenta) and antibody against DSB-1 (green). Yellow dashed line represents region of DSB-1 positive nuclei. Scalebar indicates 20 microns. B. Quantification of DSB-1 positive meiotic nuclei for wildtype (n = 1981) and him-3R93Y (n = 1918) mutant strains. NS indicates non-significant. C. Quantification of DSB-1 positive meiotic nuclei for syp-1 (n = 1918) and him-3R93Y;syp-1 (n = 1744) mutants. NS indicates non-significant. Statistical significance was assessed using two-tailed Fisher’s exact tests. (PDF) [file pgen.1010708.s003.pdf]

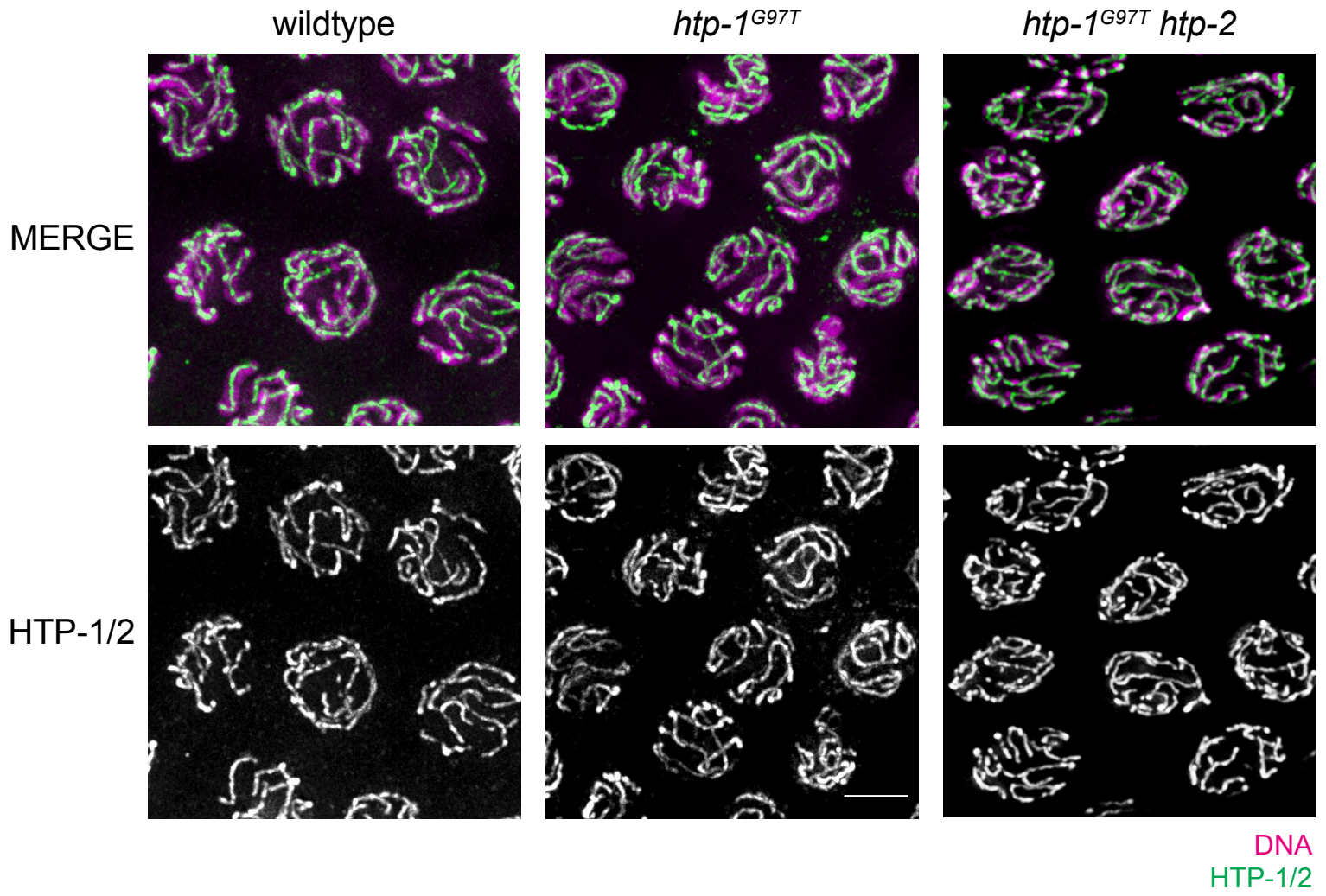

Supplement: S4 Fig — Top: Mid-pachytene nuclei from wildtype, htp-1G97T and htp-1G97T htp-2 germlines stained with DAPI (magenta) and antibody against HTP-1/2 (green). Bottom: Mid-pachytene nuclei from wildtype, htp-1G97T and htp-1G97T htp-2 germlines stained with HTP-1/2 (white). Scalebar indicates 5 microns. (PDF) [file pgen.1010708.s004.pdf]

A

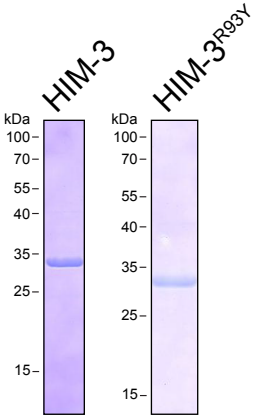

B

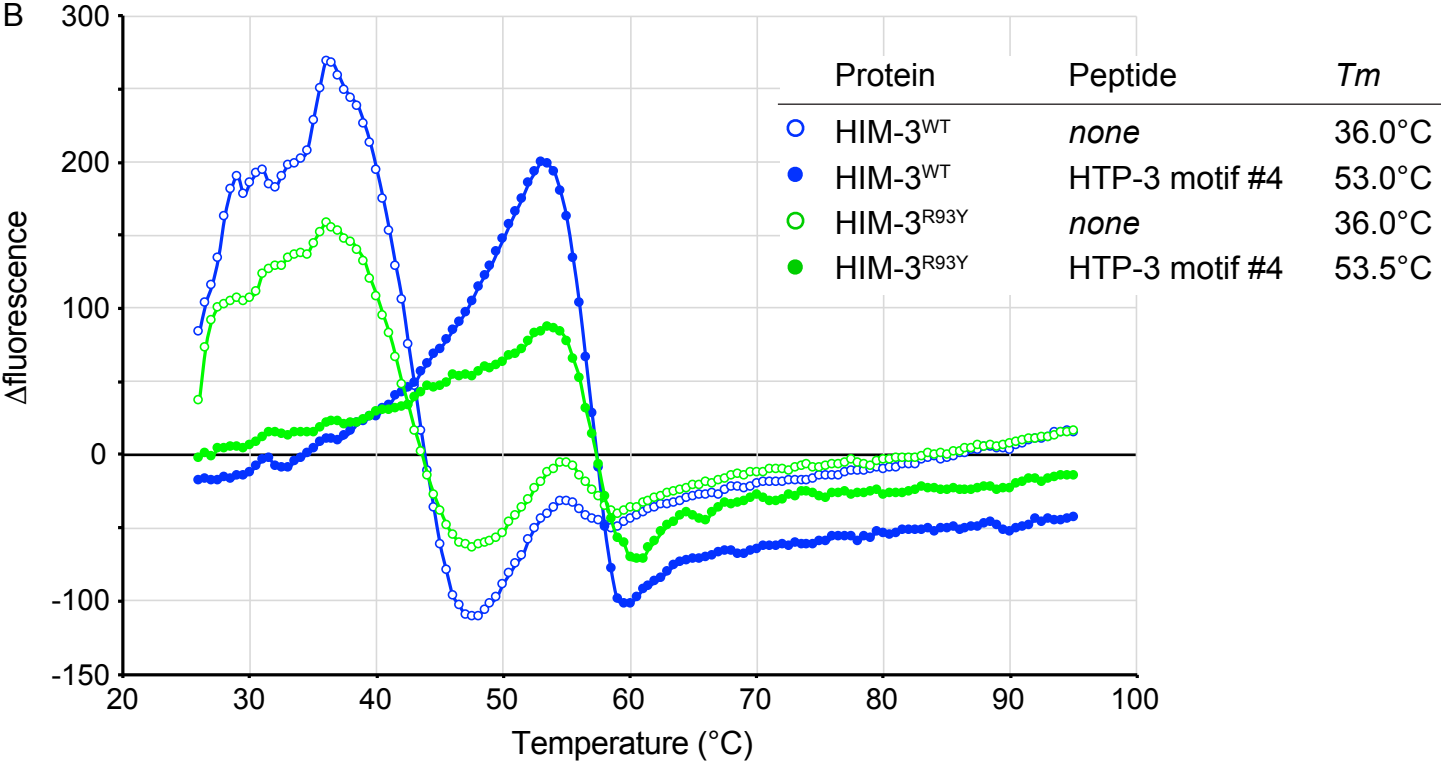

Supplement: S5 Fig — A. Coomassie stained SDS-PAGE gels of purified HIM-3 and HIM-3R93Y. B. Stability curve for purified wildtype HIM-3 (blue) and HIM-3R93Y (green). (PDF) [file pgen.1010708.s005.pdf]

A

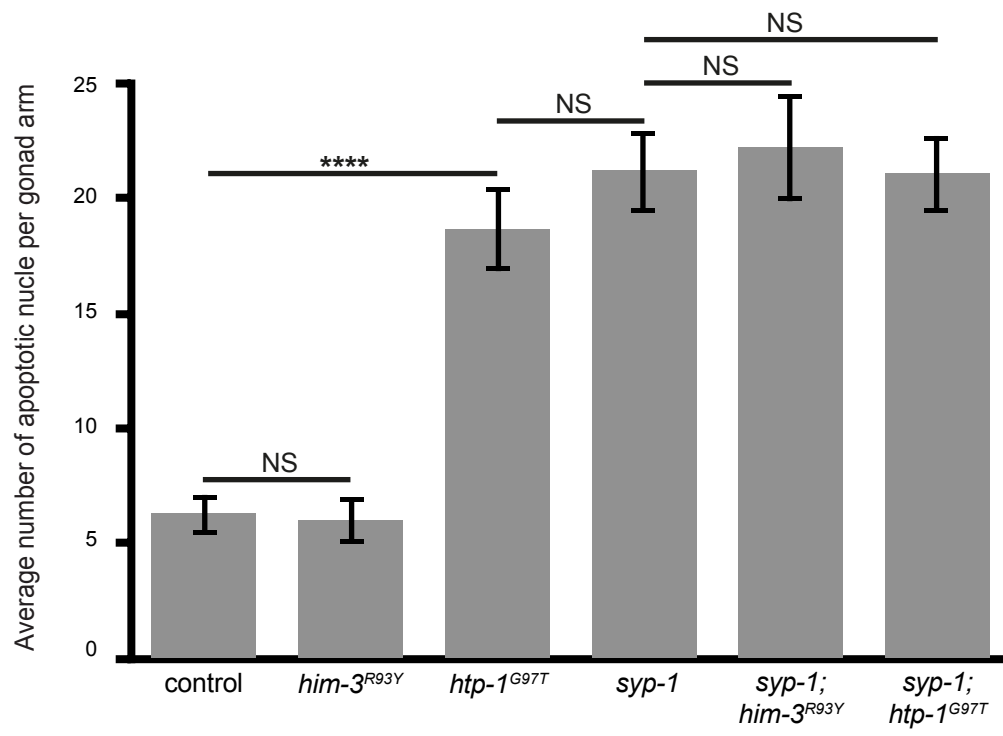

Supplement: S6 Fig — Quantification of the average number of apoptotic nuclei per germline. NS indicates non-significant and **** indicates p-value <0.0001. Statistical significance was assessed using two-tailed Student t-tests. Error bars indicate 2X standard error of the mean (SEM). (PDF) [file pgen.1010708.s006.pdf]
