# Supplementary material for: The conserved AAA ATPase PCH-2 distributes its regulation of meiotic prophase events through multiple meiotic HORMADs in C. elegans
Source: PLoS Genet. 2023 Apr 14;19(4):e1010708. doi: 10.1371/journal.pgen.1010708 (PMC10132761; doi:10.1371/journal.pgen.1010708)
Supplement: S1 Table — Table with data collection and refinement statistics for Fig 7. Explanations of equations listed below the table. (PDF) [file pgen.1010708.s007.pdf]

Table S1. Data collection and refinement statistics

	HIM-3 R93Y
**Data collection**	
Synchrotron/Beamline	ALS 8.3.1
Date collected	9/14/2018
Resolution (Å)	63.4-1.3
Wavelength (Å)	1.11583
Space Group	$P6_1$
Unit Cell Dimensions (a, b, c) Å	73.23, 73.23, 110.82
Unit cell Angles (α,β,γ) °	90, 90, 120
I/σ (last shell)	27.4
[1]R_{sym} (last shell)	0.055 (2.245)
[2]R_{meas} (last shell)	0.056 (2.081)
[3]$CC_{1/2}$ (last shell)	1 (0.677)
Completeness (last shell) %	100 (100)
Number of reflections	1,632,957
unique	82,280
Multiplicity (last shell)	19.8 (19.7)
**Refinement**	
Resolution (Å)	63.4-1.3
No. of reflections	82274
working	4255
free	78019
[4]R_{work} (last shell) (%)	14.39 (31.35)
[4]R_{free} (last shell) (%)	16.39 (32.51)
**Structure/Stereochemistry**	
No. of atoms	4093
solvent	252
hydrogen	1907
r.m.s.d. bond lengths (Å)	0.021
r.m.s.d. bond angles (°)	1.58
Ramachandran favored/allowed (%)	97.03%/100%
Molprobity score	1.55
[5]SBGrid Data Bank ID	8CXK
[6]Protein Data Bank ID	910

[1] $R_{sym} = \sum\sum_j |I_j - \langle I \rangle| / \sum I_j$, where I_j is the intensity measurement for reflection j and $\langle I \rangle$ is the mean intensity for multiply recorded reflections.

[2] $R_{meas} = \sum_h [\sqrt{(n/(n-1))} \sum_j [I_{hj} - \langle I_h \rangle] / \sum_{hj} \langle I_h \rangle$, where I_{hj} is a single intensity measurement for reflection h, $\langle I_h \rangle$ is the average intensity measurement for multiply recorded reflections, and n is the number of observations of reflection h.

[3] $CC_{1/2}$ is the Pearson correlation coefficient between the average measured intensities of two randomly-assigned half-sets of the measurements of each unique reflection.

[4] R_{work}, $R_{free} = \sum ||F_{obs}| - |F_{calc}|| / |F_{obs}|$, where the working and free R-factors are calculated using the working and free reflection sets, respectively.
