# Supplementary material for: The conserved AAA ATPase PCH-2 distributes its regulation of meiotic prophase events through multiple meiotic HORMADs in C. elegans
Source: PLoS Genet. 2023 Apr 14;19(4):e1010708. doi: 10.1371/journal.pgen.1010708 (PMC10132761; doi:10.1371/journal.pgen.1010708)
Supplement: S2 Table — (PDF) [file pgen.1010708.s008.pdf]

Table S2: Number of nuclei assayed for each genotype in each zone for all figures.

		number of nuclei in each zone					
**Figure**	**Genotype**	**1**	**2**	**3**	**4**	**5**	**6**
3C	*syp-1*	308	460	487	525	467	257
	pch-2;syp-1	470	912	882	637	358	148
	htp-3;syp-1	524	815	904	779	405	202
	pch-2;htp-2;syp-1	346	696	910	958	568	192
3E	wildtype	337	334	255	214	172	128
	pch-2	300	257	262	235	205	126
	htp-3	589	644	530	458	373	127
	pch-2;htp-3	554	568	532	457	366	128
3F	*meDf2/+*	215	202	265	263	244	188
	pch-2;meDf2/+	235	278	200	228	150	118
	htp-3;meDf2/+	533	616	450	428	344	141
	pch-2;htp-3;meDf2/+	449	438	487	425	323	149
3H	wildtype	555	679	602	542	443	262
	pch-2	326	361	361	372	262	144
	htp-3	359	430	287	349	281	184
	pch-2;htp-3	360	383	370	343	265	150
4B	*syp-1*	656	633	560	520	439	323
	pch-2;syp-1	554	541	551	485	299	210
	him-3;syp-1	602	657	584	464	339	215
	pch-2;him-3;syp-1	686	743	641	506	391	234
4D	wildtype	605	610	561	526	433	221
	pch-2	597	662	558	500	463	267
	him-3	684	801	713	636	521	263
	pch-2;him-3	415	648	508	437	357	170
4E	*meDf2/+*	310	387	327	289	405	99
	pch-2;meDf2/+	509	471	482	409	290	171
	him-3;meDf2/+	495	552	489	429	355	181
	pch-2;him-3;meDf2/+	496	602	528	431	313	194
4G	wildtype	558	683	478	480	372	203
	pch-2	596	504	518	436	362	171
	him-3	502	525	518	408	367	144
	pch-2;him-3	535	665	559	542	433	161
5B	*syp-1*	641	537	579	550	332	218
	pch-2;syp-1	508	403	466	481	283	218
	htp-1;syp-1	452	431	429	397	329	209
	pch-2;htp-1^{G97T};syp-1	550	525	556	527	336	227
5D	wildtype	545	601	589	537	383	204
	pch-2	533	517	480	557	383	240
	htp-1^{G97T}	589	644	530	458	373	127

	pch-2;htp-1^{G97T}	525	677	688	516	451	210
5F	wildtype	550	530	434	446	311	204
	pch-2	523	559	561	452	402	150
	htp-1^{G97T}	524	572	492	452	347	228
	pch-2;htp-1^{G97T}	545	516	401	375	323	118
