# Supplementary material for: The conserved AAA ATPase PCH-2 distributes its regulation of meiotic prophase events through multiple meiotic HORMADs in C. elegans
Source: PLoS Genet. 2023 Apr 14;19(4):e1010708. doi: 10.1371/journal.pgen.1010708 (PMC10132761; doi:10.1371/journal.pgen.1010708)
Supplement: Attachment — Submitted filename: ResponseToReviewers.docx [file pgen.1010708.s010.docx]

Reviewer #1: Through elegant genetic experiments, this work describes the role of PCH-2 in regulating the three essential HORMAD proteins at different stages of meiosis in C. elegans. Using a combination of pch-2 null mutants and htp-3/him-3/htp-1 loss of function mutants, the authors convincingly show an important role for PCH-2 regulation of HTP-3 during homolog pairing and synapsis, HIM-3 in recombination, and HTP-1 in meiotic progression. The authors should be commended on an extremely well written and clear manuscript- the rationale for each experiment was well described and the conclusions clearly stated. This is not trivial when working with complex genetic experiments and structural biology. In addition, the combination of genetics, cell biology, and structural biology/biochemistry is a great strength of this manuscript. My major suggestions for improving the manuscript primarily focus on strengthening the biochemical connection between PCH-2 and the HORMADs, and validating the stability of the HORMAD mutant proteins in vivo.

Major Points:

1. The experiments using a combination of mutants to show genetic interactions between PCH-2 and HTP-3/HIM-3/HTP-1 are very well done and convincingly show that PCH-2 must be involved in a pathway regulating these HORMADs. However, the major conclusion and model from this work proposes that PCH-2 directly binds to these HORMADs and changes their conformation. The Introduction and Discussion nicely justify this model: PCH-2 orthologs have been shown to perform this function in other systems. But the authors also describe how PCH-2 regulation of HORMADs may be different in C. elegans (lines 130-132, 654-657) and how the biochemistry of meiotic PCH-2/HORMADs is less convincing (lines 120-122). The assumption made by the authors that PCH-2 is directly interacting with HORMADs to change their conformation is reasonable based on evidence from other organisms, however, this manuscript would be greatly improved if this could be confirmed in C. elegans. I do not think this is a requirement for publication in PLOS Genetics- like I have stated, this assumption is reasonable based on other work and the author’s results- but I do think the authors should make a reasonable attempt to show an interaction more directly and if they cannot, to report this in the manuscript. Some experiments that may accomplish this include (but are not limited to): Immunoprecipitation of either PCH-2 or the individual HORMADs to show PCH-2/HORMAD is at least in complex together in worm lysate, direct binding experiments between purified PCH-2 and HORMADs, or mutants disrupting the region on PCH-2 that binds to HORMADs (if this region has been identified biochemically in other systems and is conserved). If these experiments are not possible because of technical limitations or could not be performed in a reasonable amount of time, the authors should state this in the manuscript and attempt to temper some of the conclusions accordingly; for example, try to avoid using terms like “acts on” (line 237) or “remodels” (line 139) when reporting conclusions if an interaction is only assumed and not directly shown.

**We thank the reviewer for their appreciation of our work. We agree that a more direct demonstration of the interaction between PCH-2 and meiotic HORMADs in *C. elegans* would enhance this story. Unfortunately, 1) the C terminally tagged PCH-2 that we generated for out studies in mitosis (published in 2015) is not functional for meiosis and the analysis of protein-protein interactions with meiotic HORMADs; 2) two hybrid experiments to test for an interaction between PCH-2 and meiotic HORMADs, were unsuccessful, even in the presence of PCH-2’s co-factor, CMT-1; and 3)  in vitro attempts to test for direct interactions between PCH-2 and meiotic HORMADs were also unsuccessful. We have changed a sentence in our introduction to emphasize this point: “In contrast to Mad2 and Rev7, attempts to undertake similar structural and biochemical analysis of PCH-2/TRIP13 and meiotic HORMADs have been less informative due to technical difficulties.”**

**Further, while the N-termini of meiotic HORMADs appears to be important for the ability to interact with, and presumably be remodeled by, PCH-2 in mice, the N-termini of meiotic HORMADs plays additional regulatory roles in *C. elegans* (Ferrandiz, 2020 and our unpublished data) and is unlikely to only affect interactions with PCH-2, potentially confounding our ability to properly interpret these experiments. This is why we used SIM to overcome these challenges. We have changed the language in the manuscript to reflect that these interactions are inferred but not demonstrated in *C. elegans*.**

2. The conclusion from Figure 1 that the localization of PCH-2 relative to the central element is consistent with PCH-2 interacting with HORMADs would be strengthened if direct co-localization between PCH-2 and HTP-3/HIM-3/HTP-1 could be shown. In addition, this would help support the conclusion of an interaction between PCH-2 and the HORMADs, as I discussed in Major Point 1. I fully appreciate that this is not always possible based on the availability of antibodies (ie if the two antibodies were raised in the same animal), but from reading the methods, it appears that a combination of PCH-2 (rabbit) and at least HTP-3 (guinea pig or chicken) may be possible. If a combination of PCH-2 and HIM-3/HTP-1 is not possible, this should be stated. In addition, showing co-localization between PCH-2 and the different HORMADs throughout meiotic progression may help inform the model that the HORMADs are acted upon by PCH-2 at different stages of meiosis.

**The images with PCH-2 and SYP-1 that were provided in the original manuscript were generated during a site visit to test out a SIM microscope and we currently do not have access to the same microscope at UCSC. In addition, we were unable to perform superresolution microscopy with both PCH-2 and HIM-3 or PCH-2 and HTP-3. For PCH-2 and HIM-3, both of the antibodies for these proteins were generated in rabbit, preventing us from performing co-staining. For PCH-2 and HTP-3, we collaborated with a faculty member here at UCSC, Dr. Sara Abrahamsson, to use her SIM microscope to colocalize PCH-2 and HTP-3. However, while we were able to recapitulate our result that PCH-2 staining is wider than SYP-1, sometimes splitting as parallel tracks, we could not achieve the same resolution with HTP-3 since it was imaged in a different wavelength that limited the extent to which data reconstruction could increase the resolution of the widefield images. While we agree that these images would have strengthened the manuscript, we propose that the provided images, combined with the magnifications and line scans demonstrating the greater width of the PCH-2 signal, support our claim that PCH-2 could remodel meiotic HORMADs on synapsed chromosomes.**

3. The authors either cite previous work or show localization of mutant HTP-3/HIM-3/HTP-1 (Figs S2, S4) to meiotic chromatin as evidence that these mutant proteins are abundant and stable. They also nicely show that mutant HIM-3 is stable in vitro (Figure S5). However, it is still important to show the abundance of these mutant proteins in worm lysate to rule out any dosage effects on the observed phenotypes. It is possible that these mutations make a particular conformation or population (i.e. the diffusive nuclei population mentioned on lines 173-174) unstable, which would lead to a different conclusion than if the protein functions normally except the mutated region. This is especially important for HTP-1 which the authors explain is susceptible to instability. This could be accomplished with a quantitative western blot comparing the relative abundance of these proteins in wild type control worm lysate and mutant worm lysate.

**Unfortunately, we do not have access to affinity purified antibodies to assess protein levels and our attempts to perform these experiments with non affinity-purified antibodies were not successful in visualizing the proteins. Moreover, while we agree that these experiments would be useful, we point out that in other reports, the absence of HTP-1 produces an acceleration of meiotic prophase, inconsistent with the phenotypes observed with *htp-1G^97T^* mutants.**

Minor points:

1. Please expand the Methods sub-section “Protein Expression and Purification” to include relevant buffers (lysis buffer, elution buffers, final buffer pure protein is in). This will make it possible to replicate these results. Also, if possible, please include a final gel of the purified protein that was used in the biochemistry experiments (Figs 7 and S5).

**We have expanded the methods to include the requested details. We have also added a new panel to Figure S5 with Coomassie-stained SDS-PAGE gels of purified HIM-3 and HIM-3^R93Y^.**

2. In lines 270-271, a very brief explanation of why C. elegans have relatively low RAD51 foci numbers compared to mammals would be appreciated for readers who are less familiar with the worm system. In mammalian spermatocytes, there are hundreds of RAD51 foci per nucleus, so it is surprising that worm oocytes only contain ~2 per nucleus.

**We have included this sentence in the section where we first discuss quantifying RAD-51 foci: “In stark contrast to what has been observed in yeast, mice and plants, *C, elegans* have substantially fewer RAD-51 foci and the number of these foci peak later in meiotic prophase [46].” As far as we know, there isn’t a clear scientific consensus on why *C. elegans* have fewer RAD-51 foci, although it likely is linked to the ability of *C. elegans* chromosomes to pair and synapse even in the absence of double strand breaks.**

3. I am confused by the error bars in Figure 4K- the text states that the authors observe no univalents in wild type and pch-2; him-3R93Y, but there are significant error bars. Perhaps my understanding of statistics is failing me, but consider including an explanation?

**We apologize and have removed these error bars.**

4. Is it possible to show a full structure of HTP-1 to include with Figure 2E, like what is shown in Figs 2C and D? It would be nice to have the inset in context with the whole protein.

**We have updated Figure 2E to include a full structure of the HTP-1 HORMA domain bound to a closure motif of HTP-3 (PDB ID 4TZQ)**

5. The peptide binding assays shown in Fig 7 are beautifully done. Is it possible to expand this assay with additional purified HORMADs or with more closure motif peptides? I appreciate that this mechanism is likely generalizable but more examples would strengthen this conclusion.

**This assay is modified from a prior manuscript (Kim et al. *Developmental Cell* 2014) in which we performed a range of binding assays with different closure motif peptides and HORMA domains. In that work, we showed that HIM-3 binds the four central closure motifs of HTP-3 (which are near-identical; we only tested one) but not other closure motifs from different HORMADs. Therefore, we don’t feel it would be beneficial to test the mutant HIM-3 HORMA domain with other peptides.**

**We agree that testing other HORMA domains would be beneficial, in theory. Unfortunately, we have never been able to purify the HTP-3 HORMA domain, precluding our ability to test the effects of the HTP-3^H96Y^ mutant. HTP-1 and HTP-2 are extremely difficult to purify, requiring coexpression with HTP-3 followed by purification away from the bound HTP-3. Given this difficulty and the likelihood that this purification method would fail for HTP-1 constructs with reduced affinity for closure motifs (like the G97T mutant), we decided not to purify these constructs.**

6. On lines 662-663 of the Discussion, the authors mention PCH-2 may reduce overall levels of HORMADs to modulate meiotic events. Could a Western blot from the pch-2 mutants show this change in HORMAD abundance?

**We should have been more clear here. We hypothesize that this change in HORMAD abundance is occurring on chromosomes and a Western blot would not report on it. We have changed this sentence to “these phenotypes suggest that PCH-2 either reduces overall levels of HORMADs on chromosomes or increases their association-dissociation dynamics to modulate these key events.”**

7. Please expand on the “IF, Antibodies and Microscopy” Methods sub-section. More details including incubation times, relevant buffers, fixation times, etc. This would help others replicate these results.

**We have expanded the methods to include the requested details.**

Reviewer #2: In this paper, Russo and colleagues show that the AAA+ ATPase PCH-2 exerts its regulatory control over several critical events in meiotic prophase in C. elegans through its interaction with different HORMA-domain proteins of the chromosome axis. Mutations in analogous positions in the HORMA domains of three proteins (HTP-3, HIM-3, and HTP-1) are found to suppress distinct phenotypes of pch-2 single mutants, leading to the conclusion that genetic interactions between PCH-2 and these HORMA proteins are important in controlling distinct processes (pairing, synapsis, crossover recombination, and meiotic progression) in meiotic prophase. Since these mutations lower the affinity of HORMA domain proteins for their closure motifs, while the assumed activity of PCH-2 is to convert the closed form of HORMA proteins to the open form, these experiments strongly suggest the idea that PCH-2 exerts control on the above events by remodeling HORMA domain proteins. These results are contrasted with the known roles of PCH-2 orthologs in other species and their biological significance is discussed in light of several particular aspects of C. elegans meiosis (such as pairing being independent of DNA breaks). This paper sheds new light on possible functions of PCH-2 and opens a new avenue toward further mechanistic understanding of the critical roles played by HORMA-domain proteins of the meiotic chromosome axis.

Major comments:

1. The genetic evidence provided for HORMAD<->PCH-2 interaction is strong for the most part, but to interpret some of the figures it will be necessary to add a full description of the quantitation method and statistical analysis. For example, in Figure 6B, the mean is shown, but there is no indication of how many gonads were examined, which cells from the gonad were considered, and how the percentages of DSB-1-positive cells from each gonad were aggregated into the Fisher's exact test. Especially in measurements with small effect size (for example, 6B showing meiotic progression in htp-1(G97T) vs pch-2;htp-1(G97T), 81% vs 77% DSB-1 positive), a full accounting of the quantitative analysis will lend more support to the conclusions.

**To make the statistics of these experiments more transparent, we have updated and  included this information in the Materials and Methods and provided n values for all analysis, either in Table S2 or in the figure legends. Details for how DSB-1 was scored as either positive or negative for Figures 8, S1 and S3 are now described in the revised version of the methods along with how DSB-1 positive cells were aggregated in the Fisher’s Exact test.**

2. The number of biological/technical replicates should be listed for each figure, and the data underlying the summary statistics made available as per PLOS policy.

**The number of replicates is included in the Materials and Methods, the number of nuclei analyzed for each figure is provided in Table S2 or in the figure legends and the data has been provided with the resubmitted manuscript.**

3. There are some conclusions made in the text that appear to be based either on observations without statistical significance, or that have not been assessed for significance, in the corresponding Figure (for example, from line 408: "we found that there was an increase in the percent of nuclei with univalents in pch-2 single mutants compared to wildtype controls…" but the figure 4K says it's not significant). This is an issue for several of the graph panels of Figures 3, 4, and 5. If there is little statistical significance to some results, then the conclusions should be given appropriate caveats.

**Despite the lack of statistical significance, the presence of univalents in some genotypes and not others is still biologically significant. To avoid this confusion, we have added this sentence: “While this difference is not statistically significant, it is biologically significant, given that we never see univalents in wildtype oocytes.”**

**In the case of other examples where the lack of significance is highlighted in panels, this is typically to demonstrate the lack of a genetic interaction (Figures 4B, 5B, 5H and 5J), which is discussed in the text.**

4. I also have a question about a point of logic in the paper, concerning how the apparently disparate genetic interactions of pch-2 with htp-3(H96Y) and him-3(R93Y) are treated. The fact that htp-3(H96Y) rescues the early synapsis phenotype of pch-2 mutants has a straightforward interpretation: the H to Y mutation makes HTP-3 more readily transition from a closed to open state (which pch-2 is normally required for). If this were the mode of action of PCH-2 on HIM-3, however, then the him-3R93Y single mutation should not phenocopy pch-2 mutations (Fig. 4I), but be more similar to wildtype, as is the case with the single htp-3(H96Y) mutation. Currently the text says that "these two alleles genetically suppress each other in the context of crossover recombination" (line 414), but in the Discussion, the mode of PCH-2 action on HTP-3 and HIM-3 are not distinguished (line 725-6). If pch-2 works by opening HIM-3, and the mutant has lower affinity for closure motifs (i.e. is more likely to adopt the open confirmation), it is not clear to me how these alleles could suppress each other. Some plausible explanation of this should be offered in the Discussion.

**We were also surprised that the *him-3^R93Y^* mutants phenocopied the *pch-2* mutation in both accelerating synapsis and affecting crossover assurance. We similarly assumed that it would behave like the *htp-3 ^R93Y^* mutant in the context of synapsis. However, *htp-3 ^R93Y^* mutants also phenocopy other aspects of *pch-2*’s mutant phenotypes, specifically its role in checkpoint induced apoptosis. What these data imply is that the open to closed cycle of meiotic HORMADs may have more far reaching implications for meiotic events than their regulation by PCH-2, as this reviewer pointed out in a later comment. In this manuscript, we limited our analysis to how meiotic events were affected by interactions with *pch-2* mutants. But it is likely that these single mutants may also provide additional information about the regulation of meiotic events like synapsis and crossover assurance.**

Minor points that should be clarified or considered:

- In lines 97–113, from the description given it is not clear to me how Hop1 in solution is supposed to be conceived -- in the "closed" conformation does it bind to a binding partner (line 97:"In the closed conformation, a peptide on a binding partner called the 'closure motif' binds the core of the HORMAD’s HORMA domain.") or to itself?

**In the described experiments, Hop1 in solution is in the closed conformation and bound to peptide fragment containing a closure motif from a binding partner, Red1. We have added this info to the description of this experiment: “While stable, open conformers of meiotic HORMADs have not been observed *in vitro* or *in vivo*, the budding yeast meiotic HORMAD, Hop1, exists in solution as two distinct conformations that are in equilibrium: the closed conformation, bound to a peptide fragment containing a closure motif from an interacting protein, and an intermediate “extended” state that has been referred to as unbuckled [28].” As discussed in the Discussion, when in the cytoplasm, Hop1 is likely bound to its own closure motif and this structure has to be remodeled by Pch2 to facilitate nuclear import and association with meiotic chromosomes: “Meiotic HORMADs in most organisms adopt a “self-closed” conformation with their HORMA domains bound to a closure motif in their own C-terminus. In budding yeast and plants, Pch2/TRIP13’s remodeling activity is needed to convert these “self-closed” HORMADs into an “open” or “unbuckled” conformation to promote their nuclear import and association with chromosomes”**

- Line 232: htp-3(H96Y) is said here to have "accelerated desynapsis", but desynapsis looks delayed instead of accelerated in Fig. 3E (pos 5,6 have higher % of nuclei with full synapsis than WT). Further, the reference given for previous report of this is #42, but ref. 42 does not refer to this.

**We apologize for the error but wildtype and *htp-3* were mislabeled in this graph. As reported in Couteau and Zetka, *htp-3^R96Y^* mutants show premature synaptonemal complex disassembly (desynapsis) in late meiotic prophase.**

- Line 393: the text says "Less than 6" (COSA-1 foci) but Fig 4I says just "5"; please clarify whether the measurements here and in Fig. is for exactly 5 foci or "5 or fewer" foci.

**We have corrected this in the text so that it is consistent with Figure 4I.**

- Please address the seeming discrepancy between the observations that RAD-51 foci are higher in the him-3R93Y mutant (Fig. 4G), while COSA-1 foci are lower (Fig. 4I) — i.e., why do higher DSB levels coexist with loss of CO assurance?

**It’s not clear why the higher DSB levels coexist with loss of CO assurance in *him-3^R93Y^* mutants, particularly since we also see higher DSB levels in *pch-2;him-3^R93Y^* double mutants and they do not exhibit a loss of crossover assurance, suggesting that these phenotypes can be uncoupled. There has been a recent report that DSBs earlier in meiotic prophase in *C. elegans* do not become crossovers (Hicks, 2022). These higher DSBs levels in *him-3^R93Y^* and *pch-2;him-3^R93Y^* mutants occur earlier than in wildtype, raising the possibility that these higher DSB levels are related to this phenomenon.**

- Finally, while the paper views HORMA domain protein function mainly through the lens of PCH-2 interaction, many of the non-PCH-2-related phenotypes seen in these novel point mutants are quite interesting in their own right and should not be deemphasized, as they provide more evidence for the idea that distinct meiotic functions are delegated to distinct HORMA domain proteins. The authors might wish to consider summarizing the PCH-2-dependent and -independent roles found for each HORMA protein in a table or chart.

**We have generated this table, included it as Table 2 and refer to it in the text.**

Reviewer #3: In the study titled “The conserved AAA-ATPase PCH-2 distributes its regulation of meiotic prophase events by remodeling multiple meiotic HORMADs in C. elegans” Russo et al. show that pch-2, the pachytene checkpoint homolog, genetically interacts with specific alleles of the three HORMADs (point mutations in the HORMA domain), htp-3, him-3 and htp-1 in different contexts during meiotic prophase in C. elegans. The authors determine that the specific alleles tested for each of the HORMAD proteins result in unique genetic interactions with pch-2. Biochemically, the authors test whether these genetic interactions affect the binding ability of the HORMAD proteins by assaying HIM-3R93Y's binding to the closure motif on HTP-3 in vitro. The authors conclude that PCH-2 remodels each of the individual HORMADs to regulate distinct meiotic functions and propose that PCH-2 remodels meiotic HORMADs from a closed to unbuckled conformation to control the progress of pairing, synapsis, meiotic recombination, and cell cycle progression.

While the ability to determine the specific role of PCH-2 in mediating its vast set of functions during meiotic prophase is important, the data presented in the manuscript falls short of the conclusion that PCH-2 remodels or regulates the HORMAD proteins. Instead, what the authors can conclude is that there is a genetic interaction between the HORMAD protein alleles and pch-2, which may very well be indirect in facilitating the final outcomes assayed, since the biochemical assay used in the manuscript does not test the role of PCH-2 in mediating the interaction between the closure motif on HTP-3 and the HIM-3R93Y allele for example. The authors make several over interpretations with very strong conclusions that are not supported by the evidence presented. The conclusions and interpretations should be dialed back to align with the data. Additionally, there is overall a lack of scientific precision in methods presented, legends, and some experiments lack of control groups (e.g., htp-1 G97T on meiotic progression, no pch-2 alone). We provide detailed comments below.

Major comments:

1. Usage of the word remodeling or regulation of HORMADs by PCH-2 is an overstatement and misleading, including in the title of the manuscript. The experiments conducted in the manuscript do not test whether PCH-2 remodels the three HORMAD domain mutant alleles. Instead, they test whether pch-2 mutant genetically interacts with these alleles. As stated above, the genetic interactions may well be indirect, which the authors acknowledge in the Discussion portion of the manuscript: Line 662: “PCH-2 either reduces overall levels of HORMADs or increases their association-dissociation dynamics to modulate these key events”; Line 673-675: “An attractive but still speculative extension to this model is that PCH-2’s remodeling of meiotic HORMADs destabilizes HORMAD-partner protein interactions”; Line 718-719, “PCH-2’s remodeling of HTP-3 and HIM-3 may indirectly result in the removal, and remodeling, of HTP-1, contributing to the regulation of meiotic progression”. The conclusions and title should be based on direct rather than speculative analysis.

**Given the evidence in other systems that PCH-2 directly interacts with meiotic HORMADs, we think it is reasonable to infer that these interactions are direct. However, as mentioned in response to Reviewer #1, we have changed the language in the manuscript to reflect that these interactions are inferred but not demonstrated in *C. elegans*.**

2. Methods section requires more details. For example, the number of replicates for each experiment needs to be provided. Method used for analysis of number of germlines per experiment states that “a minimum of three germlines” were used per genotype; three is a very small number, leading to the question of how the statistics were generated. Number of germ cells analyzed for each of the assays of pairing, synapsis, DSB, etc need to be presented per experiment. Details on measurement of percent viability and percent male self-progeny analysis needs to be provided. Overall, the details provided in the methods section are minimal which renders the manuscript scientifically less rigorous in its current state.

**To make the statistics of these experiments more transparent, we have updated and  included this information in the Materials and Methods and provided n values for all analysis, either in Table S2 or in the figure legends.**

3. Line 161: The overlapping nature of the SYPs and PCH-2 immunostaining has been published by the authors previously. Since the goal of the study is to test whether PCH-2 can reside on or close to axial elements of synapsed chromosomes and remodel meiotic HORMADs on chromosomes, analysis of localizations between the HORMADs with PCH-2, rather than the SYPs, may be more informative.

**The images with PCH-2 and SYP-1 that were provided in the original manuscript were generated during a site visit to test out a SIM microscope and we currently do not have access to the same microscope at UCSC. In addition, we were unable to perform superresolution microscopy with both PCH-2 and HIM-3 or PCH-2 and HTP-3. For PCH-2 and HIM-3, both of the antibodies for these proteins were generated in rabbit, preventing us from performing co-staining. For PCH-2 and HTP-3, we collaborated with a faculty member here at UCSC, Dr. Sara Abrahamsson, to use her SIM microscope to colocalize PCH-2 and HTP-3. However, while we were able to recapitulate our result that PCH-2 staining is wider than SYP-1, sometimes splitting as parallel tracks, we could not achieve the same resolution with HTP-3 since it was imaged in a different wavelength that limited the extent to which data reconstruction could increase the resolution of the widefield images. While we agree that these images would have strengthened the manuscript, we propose that the provided images, combined with the magnifications and line scans demonstrating the greater width of the PCH-2 signal, support our claim that PCH-2 could remodel meiotic HORMADs on synapsed chromosomes.**

4. Counting of RAD-51 foci (Fig. 3G, H and others): Almost all of the nuclei shown in the representative image (genotype and region not labeled) present with more than 3 foci, yet none of the groups on the graphs represent foci >2.5. This data needs to be better reconciled with the images shown.

**The number of RAD-51 data varies widely, particularly in zone 4, where wildtype nuclei can have anywhere from 0-9 RAD-51 foci. For ease of comparison, we have represented this analysis as average number of RAD-51 foci per nucleus, which may not entirely represent this range or the representative image. As per PloS Genetics policy, we have provided the raw data for this reviewer to assess.**

5. In Line 271, the authors state that there was “an average of 2 per nuclei” yet the figure does not show this. Similarly the error bars (SEM) do not capture the spread of the data points. In addition, previously, the authors showed ~4 foci of RAD-51 at zone 4 for wild type germlines (Fig. 4B, ref 3), while in the analysis in the current study this number seems to be far lower in the graphs. This is an important difference considering the very subtle difference in wild type and mutant germlines, a change in 2 foci/nuclei in wild type could cause error in data interpretation.

**The reviewer is correct that there is a great deal of variability in monitoring RAD-51 foci between experiments. Because of this variability, we always include wildtype worms in our individual experiments to ensure that the comparisons and conclusions we draw are rigorous, particularly given the subtle phenotypes we observe.**

6. Percent DSB-1 positive meiotic nuclei: i) There is no pch-2 single group to compare with. And ii) the difference between htp-1G97T and pch-2; htp-1G97T shown in the graph (Fig 6B) is very subtle. Given the small difference in the data, a greater number of germlines will need to assayed to assess robust statistical significance.

**We previously reported no defect in meiotic progression in *pch-2* mutants as visualized with antibodies against phosphorylated SUN-1 (Deshong, 2014). Since SUN-1 phosphorylation correlates with DSB-1 and DSB-2 localization, we included this sentence in this section: “We previously reported that *pch-2* mutants do not exhibit defects in meiotic progression despite the acceleration of pairing, synapsis and DNA repair in these mutants.”**

**We provide details on how we assessed significance between genotypes in our analysis of DSB-1 positive nuclei in the Materials and Methods section.**

Minor comments:

Figure 2 should follow Figure 1 and be proceeded by Figure 3 in the writing. As written, Figure 2 appears after Figure 3.

**We refer to Figure 2 before Figure 3 in the manuscript at line 193.**

Fig. 1 B: Please provide the genotype

**We’ve included the genotype in the Figure legend.**

Fig. 3B, D, G, I; Fig 4A, C, F, H, J; Fig. 5A, C, E, G, I: Genotype and zone numbers need to be labeled. The authors do not present representative images of defects from all the genotypes assayed and instead use one genotype on the left panel for each one of these figures. Unfortunately, this form of representation does not add any value. If the point of the single image is to show how the germ cell defect appears and how the counting is done, showing it once in the beginning would serve the purpose.

**We have added the genotype and zone numbers for the images. We disagree that these images do not add any value and have kept them in the figures to demonstrate the experimental variability we observe.**

Line 187: “Having established that PCH-2’s localization is consistent with the ability to remodel meiotic HORMADs on meiotic chromosomes, we looked for genetic interactions that might support this possibility.”- is not accurate, since the data only suggests a possible proximity between PCH-2 and HORMADs, which at the resolution tested is not indicative of remodeling.

**We do not agree.** **Given the technical limitations in demonstrating a direct relationship biochemical relationship between PCH-2 and meiotic HORMADs  (see response to Reviewer #1), this cytological analysis allows us to demonstrate that PCH-2 localizes to specific regions of meiotic chromosomes, consistent with a role in remodeling meiotic HORMADs on meiotic axes.**

Line 237: “interacts with” rather than “acts on” would be more accurate.

**We have changed this.**

Line 634: needs a reference.

**We have added the reference.**

Line 638: needs a reference.

**We have added the reference.**

Line 696-698: This statement is inaccurate. The role of HTP-1 in regulating meiotic progression is well documented in the ref [53 and 72]. In addition, in the ref 72, the authors demonstrate that phosphorylation of HTP-1 regulates synapsis formation and maintenance which in turn regulates meiotic progression. Thus, HTP-1-mediated effect on synapsis is not “indirect” and is the consequence of defects in synapsis formation and maintenance due to lack of HTP-1 phosphorylation.

**We have removed the term indirect from this sentence.**

Line 771-772: Please see the above comment.

**We disagree with the interpretation that HTP-1’s role in regulating synapsis and recombination is direct and favor the interpretation that it is indirect as a consequence of a primary role in regulating meiotic progression.**

Line 719: Omit the comma.

**We have removed the comma.**

Line 1429: Since the manuscript does not address how PCH-2 may act to remodel the different meiotic HORMADs the title of the model figure should be altered to more accurately reflect data presented on the interactions of pch-2 with the hormads.

**Based on comments from reviewer 1 and this reviewer, we have removed the term remodeling from the title of the paper. However, the proposed model and its title reflects our interpretation of the data and thus, we have not changed it.**
